# Supplementary material for: A Critical View of the Application of the APEX Software (Aqueous Photochemistry of Environmentally-Occurring Xenobiotics) to Predict Photoreaction Kinetics in Surface Freshwaters
Source: Molecules. 2019 Dec 18;25(1):9. doi: 10.3390/molecules25010009 (PMC7017383; doi:10.3390/molecules25010009)
Supplement: Supplementary file 1 [file molecules-25-00009-s001.zip › 3.2.4_gcc-4.4.0.zip!/3.2.4_gcc-4.4.0/3.2.4_gcc-4.4.0/doc/octave/html/interpreter/Error-bar-series.html]

Error bar series - Untitled


Next: Line series,
Previous: Contour groups,
Up: Object Groups


---

##### 15.2.8.5 Error bar series

Error bar series are created by the `errorbar` function. Each
`hggroup` element contains two line objects representing the data and
the errorbars separately. The properties of the error bar series are

`color`: The RGB color or color name of the line objects of the error bars. See Colors. `linewidth` `linestyle`: The line width and style of the line objects of the error bars. See Line Styles. `marker` `markeredgecolor` `markerfacecolor` `markersize`: The line and fill color of the markers on the error bars. See Colors. `xdata` `ydata` `ldata` `udata` `xldata` `xudata`: The original x, y, l, u, xl, xu data of the error bars. `xdatasource` `ydatasource` `ldatasource` `udatasource` `xldatasource` `xudatasource`: Data source variables.
